# Transient early preeclampsia in twin pregnancy with a triploid fetus: a case report

**DOI:** 10.1186/1752-1947-3-7311

**Published:** 2009-05-26

**Authors:** Clasien van der Houwen, Tineke Schukken, Mariëlle van Pampus

**Affiliations:** 1 Department of Obstetrics and Gynecology, Tjongerschans Hospital Heerenveen, Thialfweg, 8441 PW Heerenveen, The Netherlands; 2 Department of Obstetrics and Gynecology, University Medical Centre, 9700 RB Groningen, The Netherlands

## Abstract

**Introduction:**

Triploid pregnancies have an increased risk of early preeclampsia. Twin pregnancies consisting of one healthy fetus and one complete or partial molar, with or without a triploid fetus, are rare and management is complex.

**Case presentation:**

A 33-year-old Caucasian woman presented with a dichorionic diamniotic twin pregnancy. One fetus showed early growth restriction resulting in fetal death at 20 weeks. The placenta was enlarged with some cysts. Chorionic villus biopsy confirmed triploidy. At 21 weeks, the patient developed preeclampsia with a blood pressure of 154/98 mmHg and proteinuria (24 hour protein excretion of 2.5 g/L), for which she was hospitalized. Without pharmacological interventions, the blood pressure normalized and proteinuria disappeared. At 35 weeks, she again developed preeclampsia. A cesarean section was performed at 38 weeks and a healthy child was born.

**Conclusions:**

Survival of the healthy fetus is possible in a twin pregnancy with a triploid fetus complicated by early preeclampsia. The pregnancy should not be terminated if the triploid twin has died and as long as conservative management is safe.

## Introduction

Triploidy is a genetic disorder with an extra haploid set of chromosomes resulting in a total of 69 chromosomes. Two types of triploidy can be distinguished according to the parental origin [1]. Type I, with the additional chromosome set being of paternal origin (diandric), is consistent with normal growth of the fetus, with increased nuchal translucency, and an enlarged and partially multicystic placenta with elevated levels of maternal serum beta human chorionic gonadotropin (β-hCG). Partial molar gestations are usually associated with triploidy of diandric origin.

Type II, with the additional chromosome set being of maternal origin (digynic), is characterized by a small but normal placenta with decreased levels of β-hCG and asymmetrical fetal growth restriction. Common structural defects in both types are malformed hands, head, heart and face [2].

Triploid pregnancies rarely advance into the second trimester, but if they do, a high risk of early onset severe preeclampsia is noticed as a result of the molar tissue of type I triploidy. In a series of 17 triploid pregnancies, six cases (35%) developed early preeclampsia or hypertension [3]. Elevated serum β-hCG levels and placentomegaly were associated with a higher risk of preeclampsia but with low levels of β-hCG, there was no association.

Twin pregnancies consisting of one healthy fetus and one complete or partial molar, with or without a triploid fetus, are rare. The molar tissue can provoke early preeclampsia, heavy vaginal bleeding and persistent gestational trophoblastic disease (pGTD). Experiences with partial molar twin pregnancies are limited. Only five cases of a triploid fetus and a healthy co-twin have been reported [4]-[8]. Of these five cases, two co-twins survived after selective abortion of the triploid fetus. In a large series of 77 complete molar twin pregnancies, 40% successful outcome for the healthy co-twin was reported for parents who wished to continue their pregnancy [9]. Neither serious obstetric complications nor an increase in development of pGTD was noticed. Three pregnancies were terminated because of preeclampsia.

Partial molar pregnancies are rarely associated with persistent or metastatic disease, but early preeclampsia is often reported. In three cases [4]-[6] of five concerning a triploid fetus and a healthy co-twin, a therapeutic abortion was performed, two of which were for severe preeclampsia. There are only two cases reported with survival of the healthy co-twin, both after selective abortion of the triploid fetus. One healthy co-twin was born after 27 weeks' gestation and the other after 38 weeks' gestation [7,8].

Preeclampsia can occur even after the triploid fetus has died. Nugent [4] reported a case where selective termination of the triploid twin was performed at 15 weeks' gestation. At 19 weeks, severe preeclampsia developed, necessitating therapeutic abortion.

We present the first reported patient with triploid twin pregnancy with a successful outcome for the healthy co-twin after early transient preeclampsia.

## Case presentation

A 33-year-old Caucasian woman, gravida 3, para 1, was admitted to our clinic. Her obstetric history mentioned a miscarriage and a pregnancy complicated by intrauterine growth restriction, without signs of preeclampsia. At 37 weeks' gestation, cesarean section was performed because of fetal distress. A boy was delivered, weighing 2015 g, with Apgar scores of 9 and 10 at one and five minutes, respectively. Histology of the placenta revealed 10% infarctions and a thrombus in the umbilical cord. Blood analysis after three months showed no hemostatic abnormalities associated with an increased risk of thrombosis.

Ultrasound examination of the index pregnancy at 11^2^ weeks' gestation showed a dichorionic diamniotic twin pregnancy with measurements consistent with gestational age. The Crown Rump Lengths were 39 mm, consistent with 10^6^ weeks, and 45 mm, consistent with 11^2^ weeks. Nuchal translucency thickness measurements were not performed. No abnormalities of the placenta were documented.

Her blood pressure was 125/70 mmHg. The pregnancy was complicated by episodes of vaginal bleeding at 16 weeks' gestation. Ultrasound showed one fetus with normal growth and one with early growth restriction and measurements consistent with 13 weeks. An echogenic area was interpreted as blood clots. At 20 weeks' gestation, fetal death of the abnormal fetus was noticed. One week later, the patient was asymptomatic but her blood pressure increased (154/98) which prompted the suspicion of a partial molar pregnancy. An enlarged placenta of 10 cm × 12 cm with some cysts was prominent on the anterior wall. Blood flow had ceased in this placenta. Urinary protein excretion was 2.5 g/L. Maternal serum beta-human-chorionic-gonadotropin (β-hCG) was markedly raised: 423,000 IU/L. Other laboratory investigations were normal.

The patient was sent to a University Hospital because of early preeclampsia and suspicion of a triploid twin. We decided to perform a chorionic villous biopsy because the placenta of the dead fetus was on the anterior wall. We did not perform a chorionic villous biopsy of the placenta of the healthy twin because no abnormalities were noticed by ultrasound and the placenta was located on the posterior wall.

**Figure 1:**
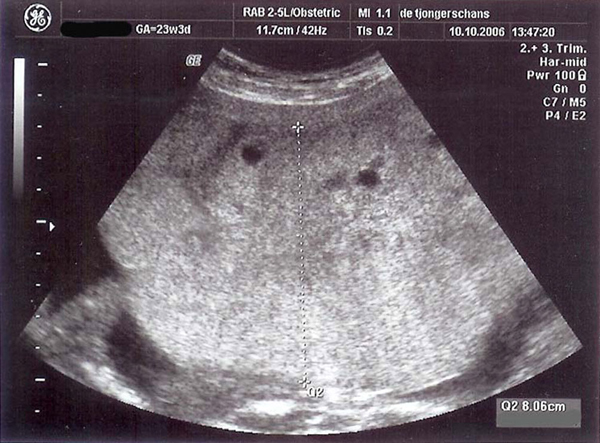
**Placenta at 23 weeks**.

Chorionic villus biopsy confirmed triploidy, 69, XXY. Without pharmacological interventions, the blood pressure stabilized, proteinuria decreased, and β-hCG decreased to 222,835 IU/L. At 22 weeks, the patient was discharged. She was reviewed twice a week. Urinary protein excretion was positive until 23 weeks, and blood pressure slowly decreased and normalized at 30 weeks. The β-hCG further decreased to 27,600 IU/L at 33 weeks. Episodes of some vaginal bleeding and cramps occurred up to 28 weeks' gestation. The placenta of the triploid twin was still enlarged until 23 weeks: 8 cm × 12 cm (Figure 1).

**Figure 2:**
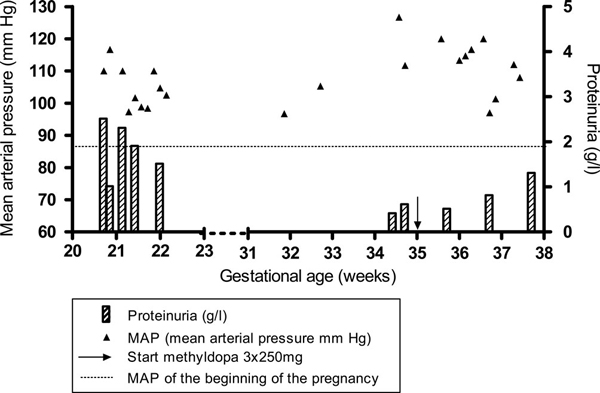
**Graphic analysis of the mean arterial blood pressure (mmHg) and proteinuria (g/24 hours)**. Urine protein stick was negative after 23 weeks. Left axis, mean arterial pressure (mmHg); right axis, proteinuria (g/L); horizontal axis, gestational age; hatched fill, proteinuria g/L; filled triangles, MAP (mean arterial pressure mmHg); arrow, start of methyl-dopa 3 × 250 mg.

At 35 weeks, the patient developed preeclampsia again and was hospitalized. Her blood pressure increased to 170/105 mmHg and proteinuria to 0.8 g/L. Methyldopa 3 × 250 mg was initiated to control the blood pressure with good result (Figure 2). Blood analysis showed no signs of hemolysis, elevated liver enzymes, low platelets (HELLP) syndrome. At 38 weeks, a cesarean section was performed for fetal distress. A healthy girl weighing 2,710 g was born with Apgar scores of 9 and 10 at one and five minutes, respectively. The placenta of the triploid fetus was necrotic and as a result of autolysis, no further histologic information on fetus and placenta were available. β-hCG follow-up showed no signs of persistent gestational trophoblastic disease (pGTD). Four days after delivery, the level had already decreased to 440 IU/L.

## Discussion

We present the first reported patient with transient preeclampsia in a twin pregnancy with a triploid fetus. In our patient, placentomegaly and high levels of β-hCG accompanied the development of preeclampsia. This is consistent with the reported series of Rijhsinghani *et al.*[3] and two other case reports of triploid twin pregnancies complicated by preeclampsia [4,6].

The presentation of the triploid fetus is not consistent with the two types of triploidy. Placentomegaly and preeclampsia are characteristics of type I triploidy, whereas growth restriction is a type II characteristic. The finding of growth restriction in a type I triploidy seems atypical but has been reported [10]. Rijsinghani *et al.* reported seven patients with triploid pregnancies who became preeclamptic. Four of them showed fetal growth restriction. The combination of growth restriction and preeclampsia in a triploid pregnancy does not seem to be uncommon. We believe growth restriction is not a specific type II characteristic.

Another interesting point is the possibility of selective abortion to improve the outcome for the healthy fetus. In retrospect, we should have considered karyotyping at 16 weeks' gestation, when intrauterine growth restriction of three weeks was noted in one fetus. After the result of triploidy, we could have subsequently offered selective abortion. In our patient, the natural death of the triploid fetus at 20 weeks undoubtedly rescued the remaining healthy fetus. Regression of preeclampsia has been reported in twin pregnancies with early preeclampsia linked to a lethal condition in one twin, and in which selective abortion was performed [11].

Early selective abortion of the triploid twin, at least before 20 weeks, is not only indicated to prevent preeclampsia but also to decrease the preterm delivery risk for the remaining fetus [12]. Two cases of successful outcome for the normal co-twin in a triploid twin pregnancy have been reported [7,8]. In both cases, selective abortion of the triploid fetus was performed.

## Conclusion

This case shows that, after the death of the triploid twin, the partial molar placenta can still cause preeclampsia, but the preeclampsia can regress. We conclude that, in the case of a twin pregnancy with a triploid fetus and early preeclampsia, there is no need for early termination of the pregnancy. As long as the preeclampsia is stable, successful outcome for the healthy co-twin is possible. If the triploid fetus is still alive, selective abortion may be offered.

## Consent

Written informed consent was obtained from the patient for publication of this case report and any accompanying images. A copy of the written consent is available for review by the Editor-in-Chief of this journal.

## Competing interests

The authors declare that they have no competing interests.

## Authors' contributions

CvdH was responsible for the patient until referral to the University Hospital and after discharge until delivery. She prepared the first draft and wrote the final manuscript. TS, as medical student, was involved in the treatment of the patient. She prepared the figure. MvP was involved in the treatment at the University Hospital. TS and MvP gave comments on the first draft. All authors approved the final manuscript.

## References

[B1] MacFadden E Kwong LC Yam IY Langlois S Prenatal origin of triploidy in human fetuses: evidence for genomic imprinting Hum Genet 1993 92 465 469 10.1007/BF00216452 7902318

[B2] Jauniaux E Brown R Rodeck C Nicolaides KH Prenatal diagnosis of triploidy during the second trimester of pregnancy Obstet Gynecol 1996 88 983 989 10.1016/S0029-7844(96)00330-4 8942839

[B3] Rijhsinghani A Yankowtz J Strauss RA Kuller JA Patil S Williamson RA Risk of preeclampsia in second-trimester triploid pregnancies Obstet Gynecol 1997 90 884 888 10.1016/S0029-7844(97)00540-1 9397095

[B4] Nugent CE Punch MR Barr M LeBlanc L Johnson MP Evans MI Persistence of partial molar placenta and severe preeclampsia after selective termination in a twin pregnancy Obstet Gynecol 1996 87 829 831 8677104

[B5] Steller MA Genest DR Bernstein MR Lage JM Goldstein DP Berkowitz RS Clinical features of multiple conception with partial or complete molar pregnancy and coexisting fetuses J Reprod Med 1994 39 147 154 8035369

[B6] Nwosu EC Ferriman E McCormack MJ Williams JH Gosden CM Partial hydatidiform mole and hypertension associated with a live fetus: variable presentation in two cases Hum Reprod 1995 10 2459 2462 8530687 10.1093/oxfordjournals.humrep.a136320

[B7] Gassner R Metzenbauer M Hafner E Vallazza U Philipp K Triploidy in a twin pregnancy: small placenta volume as an early sonographical marker Prenat Diagn 2003 23 16 20 10.1002/pd.506 12533806

[B8] Mendilcioglu I Ozcan M Bagci G Simsek M Kursun S Guven L Taskin O Triploidy in a growth discordant twin pregnancy after intracytoplasmic sperm injection treatment Fetal Diagn Ther 2006 21 65 67 10.1159/000089050 16354978

[B9] Sebire NJ Foskett M Paradinas FJ Fisher RA Francis RJ Short D Newlands ES Seckl MJ Outcome of twin pregnancies with complete hydatidiform mole and healthy co-twin Lancet 2002 359 2165 2166 10.1016/S0140-6736(02)09085-2 12090984

[B10] Stefos T Plachouras N Mari G Cosmi I lolis D A case of partial mole and atypical type I triploidy associated with severe HELLP syndrome at 18 weeks' gestation Ultrasound Obstet Gynecol 2002 20 403 404 10.1046/j.1469-0705.2002.00822.x 12383328

[B11] Heyborne KD Porreco RP Selective fetocide reverses preeclampsia in discordant twins Am J Obstet Gynecol 2004 191 477 480 10.1016/j.ajog.2004.01.009 15343224

[B12] Lynch L Berkowitz RL Stone J Alvarez M Lapinski R Preterm delivery after selective termination in twin pregnancies Obstet Gynecol 1996 87 366 369 10.1016/0029-7844(95)00455-6 8598956

